# Early clinical and metabolic response to tazemetostat in advanced relapsed INI1 negative epithelioid sarcoma

**DOI:** 10.2144/fsoa-2020-0173

**Published:** 2021-01-12

**Authors:** Ghazal Tansir, Sameer Rastogi, Shamim Ahmed Shamim, Adarsh Barwad

**Affiliations:** 1 Sarcoma Medical Oncology Clinic, BRA IRCH, All India Institute of Medical Sciences, New Delhi, India; 2 Department of Nuclear Medicine, All India Institute of Medical Sciences, New Delhi, India; 3 Department of Pathology, All India Institute of Medical Sciences, New Delhi, India

**Keywords:** epigenetics, epithelioid sarcoma, personalized medicine, targeted therapy, tazemetostat

## Abstract

Epithelioid sarcoma (ES) is a rare soft tissue sarcoma with an incidence of 0.05 per 100,000 population in the USA. It is characterized by multiple local recurrences and regional lymph nodes form the commonest site of metastases. The function of Integrase Inhibitor 1 (INI1) protein is lost in more than 90% of cases, which was the basis for the introduction of tazemetostat into the therapeutic armamentarium for management of advanced ES. The efficacy and manageable toxicity profile of tazemetostat have been demonstrated recently, leading to its accelerated approval for treatment of advanced ES. We report one of the first real-world cases of relapsed, metastatic ES treated with tazemetostat. The patient attained partial response with the therapy and is tolerating the drug well without serious toxicities.

## Lay abstract

Epithelioid sarcoma (ES) is a rare soft tissue sarcoma found in 0.05 people per 100,000 in the USA. It can recur multiple times in the lymph nodes of the involved site. ES involves the loss of the INI1 protein and this pathway is targeted by a new drug, tazemetostat. Recent studies have found this drug to be safe and effective for advanced ES. We have utilized this drug for a patient with metastatic ES who had history of multiple relapses. The patient had an encouraging response with the therapy and did not experience any serious side effects with this new drug.

Epithelioid sarcoma (ES) is a rare, slow-growing sarcoma constituting less than 1% of all soft tissue sarcomas [1]. It is a male-predominant sarcoma [2] and peaks in the second to fourth decade [3] with SEER registries reporting an incidence of 0.05 per 100,000 US population [4]. This tumor is divided into proximal and distal subtypes with the latter being the canonical subtype of the disease. The most common site of disease is in the extremities while the proximal subtype can also occur in the pelvis, perineum and genital tract [5]. The course of the disease is marked with local recurrences and 30–50% patients eventually develop distant metastases [6]. Regional lymph nodes are the most frequent site of metastases while lungs, bones and brain form the common distant metastatic locations. The outcome of patients with metastatic ES is dismal with median post-distant metastases survival of 8 months as described by Spillane *et al.* [7].

The diagnosis of ES can be a challenge due to its heterogenous morphology and rare incidence, causing it to mimic various malignant and reactive conditions. It has classically been described to express vimentin, cytokeratin (CK8, CK19, CK14), epithelial membrane antigen (EMA) and ETS-related gene (ERG). Studies reveal that ES is defined by the complete loss of SMARCB1/integrase interactor 1 (INI1) in over 90% of cases [8] and can be diagnosed by immunostaining for INI1. INI1 negativity has previously also been described in malignant rhabdoid tumors, 50% of epithelioid malignant peripheral nerve sheath tumors, synovial sarcoma, some myoepithelial carcinomas [8] and poorly differentiated chordomas [9].

The INI1 protein is a part of the SWI/SNF chromatin remodeling complex and encoded by the *SMARCB1* gene. INI1 has an interwoven role among various pathways such as p16-RB, canonical WNT, sonic hedgehog signaling and polycomb pathways, which are responsible for tumor suppression and cell differentiation. The loss of INI1 function by *SMARCB1* gene deletion, mutation or epigenetic modification [10] leads to overactivation of the histone lysine methyltransferase complex PRC2. PRC2 overactivity has been demonstrated to cause cell proliferation and silencing the genes responsible for differentiation by action of its catalytic unit, EZH2.

Literature on management of advanced ES is limited to case reports and small retrospective case series, and reveals only few options among cytotoxic and targeted therapies. Frezza *et al.* [11] described 115 patients with advanced ES on palliative chemotherapy in a multi-institutional case series and found anthracycline-based regimens were the most commonly used (74%) and yielded median progression-free survival (PFS) of 6 months. Gemcitabine-based therapy and pazopanib were the other regimens in use, with median PFS of 4 and 3 months, respectively. The response rates with anthracyclines and gemcitabine-based regimens were 22 and 27% respectively, with pazopanib having a 0% response rate. Apart from anthracyclines and gemcitabine, data on other cytotoxic chemotherapeutic agents are sparse, with few case reports describing the use of trabectedin [12], dacarbazine [13] and vinorelbine [14].

In this era of molecular profile-driven medicine, the search for newer agents for treatment of ES is ongoing. Initial preclinical data suggest that EZH2 inhibition could translate to apoptotic activity in INI-negative malignant rhabdoid tumors cells, which has opened up a therapeutic avenue in ES [15]. Tazemetostat, an oral inhibitor of the epigenetic modifier EZH2, has been found to produce durable tumor regression in preclinical setting and has been followed by clinical trials that have established its safety and efficacy [16].

A Phase I trial by Italiano *et al.* [17] established the safety and toxicity profile of tazemetostat in refractory B-cell non-Hodgkin lymphomas and advanced solid tumors including INI1-negative ES (7%). This open-label multicenter study used dose escalation from 100 mg twice daily to 1600 mg twice daily in 28-day cycles and established the recommended Phase II dosage to be 800 mg twice daily. The most common adverse events were asthenia (33%), nausea (20%), anemia (14%), vomiting (9%) and anorexia (6%). Objective tumor response was found in 5% patients with solid tumors, all of which were INI1-negative. Moreover, stable disease was found in two patients with INI1-negative ES with a prolonged duration of response (more than 20 months), which was longer than the duration noted among other solid tumors.

This was followed by a multinational Phase II open-label basket study comprising 62 INI-negative ES patients which led to the accelerated approval of tazemetostat for locally advanced or metastatic ES not amenable to complete surgical resection. The results of this trial have recently been published and tazemetostat has been reported to produce an overall response rate (ORR) of 15% with median PFS and OS of 5.5 months (95% CI: 3.4–5.9) and 19 months (11–not estimable) respectively [18]. In this case report, we describe our early experience with tazemetostat in a patient of metastatic, recurrent INI1 negative ES who attained partial response to therapy with this novel first-in-class EZH2 inhibitor.

## Case summary

**Figure 1.**
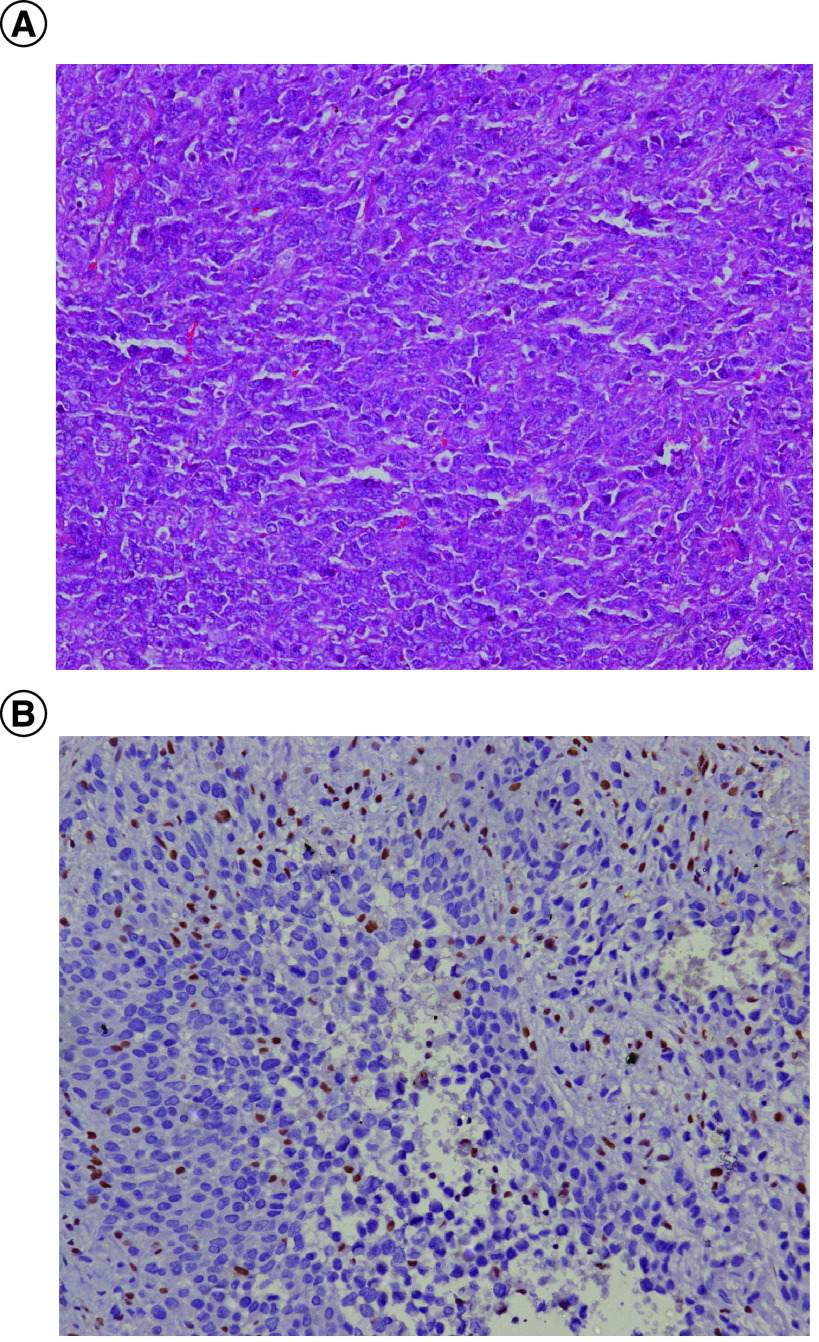
Histopathological images of the tumor with micropscopy and immunohistochemistry. **(A)** Representative histophotomicrograph of the tumor showing cells with prominent nucleoli and moderate to abundant cytoplasm arranged in sheets showing epithelioid morphology. **(B)** Immunohistochemical stain for INI1 protein showing loss of its expression compared with positive internal control.

Our patient is a 24-year-old male without comorbidities who presented in August 2017 with complaints of recurrent ulcer on their left leg for 18 months, for which he underwent surgery twice. He presented to us with 3rd local recurrence with inguinal nodal metastases. Histological examination showed a tumor with cells having epithelioid morphology, arranged in sheets and nodules with intervening fibrous septae. Immunohistochemistry showed expression of cytokeratin, vimentin and loss of INI1, establishing the diagnosis of epithelioid sarcoma (Figure 1).

He underwent wide local excision of the tumor in August 2017 with inguinal nodal dissection and adjuvant radiotherapy. After a treatment-free interval of 5 months, the patient had a local recurrence at the operated site and underwent below knee amputation in March 2018 and inguino-femoral nodal dissection for isolated nodal recurrence on June 2018.

The disease recurred after 4 months at the amputated stump, popliteal and inguinal nodes and he was started on palliative chemotherapy with doxorubicin from October 2018 to February 2019. Interim scan after three cycles of chemotherapy showed partial response while an end of treatment scan done in March 2019 was suggestive of stable disease. However, there was an inguino-femoral region nodal recurrence in July 2019 after treatment-free interval of 4 months. The patient was then started on pazopanib, with which the disease stabilized for 11 months till June 2020 when there was disease progression with cutaneous and bony metastases. The patient was symptomatic, dependent on analgesics and was facing severe limitations in performing his activities of daily living.

**Figure 2.**
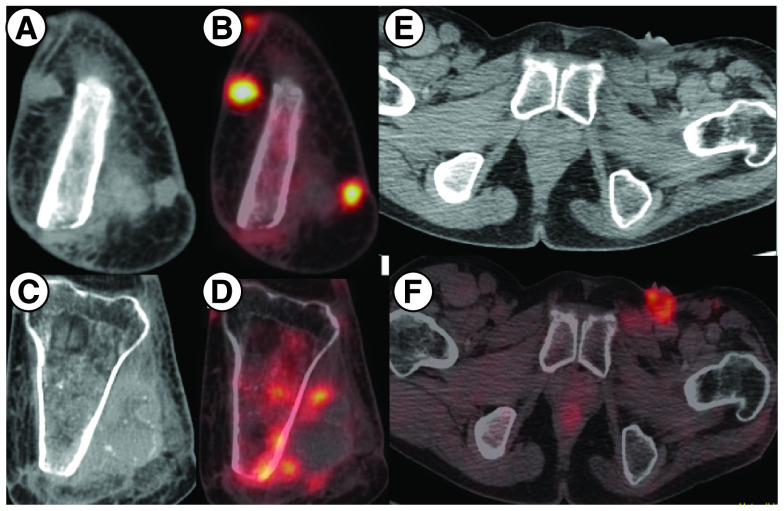
PET scan imaging showing radiographic findings prior to starting tazemetostat. **(A & B)** MIP image showing focal increased tracer uptake in the left inguinal region and the left knee joint region corresponding to cutaneous nodules at the amputated site of left tibia and fibula on CT showing increased FDG uptake on fused PET-CT. **(C & D)** Areas of sclerosis in the left tibia with increased FDG uptake. **(E–G)** Ulcerated left inguinal lymph nodes on CT showing increased FDG uptake on fused PET-CT. CT: Computed tomography; MIP: Maximum intensity projection.

**Figure 3.**
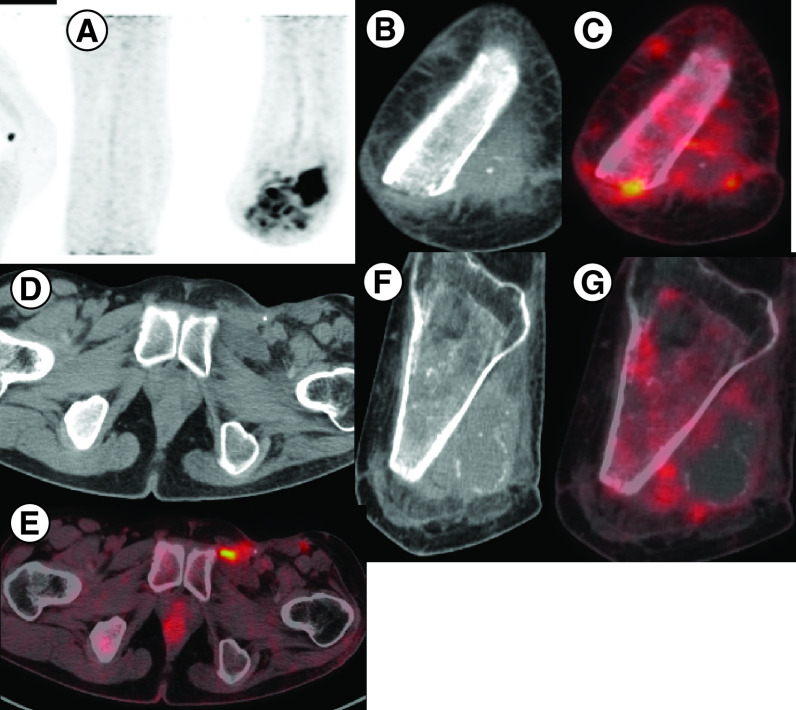
PET Scan imaging showing radiographic findings after treatment with tazemetostat. **(A)** MIP image showing focal increased tracer uptake in the left inguinal region and in the left knee joint region. **(B & C)** Previously seen cutaneous nodules in baseline image show resolution in size and metabolic activity in the present study. **(D & E)** Ulcerated subcentimetric left inguinal lymph nodes on CT showing increased FDG uptake on fused PET-CT scan. **(F & G)** Sclerosis in left tibia showing decreased FDG uptake compared with previous scan with overall findings suggest partial resolution of the disease following therapy. CT: Computed tomography; MIP: Maximum intensity projection.

Based on the results reported in the Phase II EZH2–202 trial, he was started on tazemetostat at 800 mg twice daily from July 2020. He experienced clinical benefit within 2 weeks with independence from analgesic support and resumption of routine activities. Disease assessment by PET-CT after 2 months of therapy shows resolution of in size and metabolic activity of the involved sites, suggestive of partial resolution of the disease on tazemetostat. (Figures 2 & 3). The patient is tolerating the therapy well without any grade III/IV toxicities and continues to be on regular follow-up.

## Discussion

The age of our patient lies within the age bracket classically described in literature for ES [19]. Spunt *et al.* analyzed 63 patients of ES under the age of 30 years and found 14% patients had regional lymph node metastases [20]. Similarly, a single-institution retrospective review of 20 patients by Guzzetta *et al.* showed that 90% of patients had localized disease at presentation while 10% had nodal metastatic disease [21]. Upfront surgery was performed in 95% of cases out of whom 35% (7/20) developed recurrent disease which comprised local recurrence in 57.1% (4/7) and distant metastases in the rest. However, the number of recurrences did not affect the survival outcomes and the time to recurrence did not decrease with increasing number of recurrences. Our patient also had multiple nodal recurrences separated by an interval of 4–5 months and underwent multiple surgeries with curative intent.

Radical excision with microscopically negative margins, therapeutic lymph node dissection and peri-operative radiotherapy form the mainstay of initial treatment of ES [22]. Peri-operative chemotherapy has been used in large, high-grade or metastatic tumors [23] producing responses in 0–15% patients [24–26]. Local recurrences should be evaluated by regional MRI imaging for in-transit and nodal metastases which have to be addressed along with lymph nodal dissection and radical surgery [27]. Systemic therapy in advanced ES remains poorly described, with scant prospective data for the same. Data collated by Touati *et al.* [28] from four EORTC studies showed that pazopanib prescribed in the second-line setting produced an ORR of 11% (1/9) with median PFS of 4.04 months. Our patient attained higher PFS of 11 months with second-line pazopanib compared with the previous studies.

Tazemetostat is a new treatment option in the therapeutic arsenal for this protracted and recurrent disease. It is one of the rare drugs to be given approval based upon a single-arm Phase II trial and is reflective of the growing influence of precision oncology in sarcomas. The EZH2–202 trial [18] reported an ORR of 15% including 1.6% complete response with disease control rate (DCR, the sum of the complete response, partial response and stable disease rates)26% at 32 weeks. At a median follow-up of 13.8 months, the duration of response was not reached (95% CI: 9.2–not estimable) and 27% patients were on therapy at data cutoff. About 61% of patients had received at least one treatment line, and 25% treatment-naive and 8% pretreated patients attained an objective response to tazemetostat. Our patient has received two lines of therapy, but experienced good early clinical benefit and a partial radiological response. The median time to response in the trial was 3.9 months (IQR: 1.9–7.4) while our patient responded within 2 months of therapy. The toxicity profile was predominantly grade 1–2 while grade 3–4 adverse events consisted of anemia (13%), weight loss (6%), pleural effusion (5%), reduced appetite (5%) and cancer pain (5%). In total, 3% patients had serious adverse events, including seizure (n = 1) and hemoptysis (n = 1) while one patient required dose reduction due to reduced appetite. Physicians must also be aware that tazemetostat is associated with an increased risk of secondary malignancies such as acute myeloid leukemia, T lymphoblastic lymphoma and myelodysplasia [29]. The only toxicity in our patient is grade 1 vomiting and diarrhea with no treatment related adverse effects necessitating drug interruption or dose reduction. Further follow-up is required to recognize other drug-related toxicities and to ascertain if early response translates to durable outcomes.

Newer therapeutic options are currently under study for the management of advanced ES. Dasatinib was explored by Shuetze *et al.* in a cohort of indolent sarcomas and was found to produce a 6-month PFS rate of 57% in ES; however, the 2- and 5-year OS were 21 and 0%, respectively [30]. Trials on immunotherapy have yielded interesting results with the KEYNOTE-051 study reporting one partial response to pembrolizumab [31] while Paoluzzi *et al.* have reported one partial response attained on nivolumab [32]. Results from the ongoing Phase Ib/III trial by Sen *et al.* [33] on tazemetostat combined with doxorubicin as frontline therapy for advanced ES are eagerly awaited.

Our study provides the first real-world report on early experience with tazemetostat in advanced ES in a symptomatic patient post-multiple lines of therapy. The early-onset significant clinical and radiological benefit obtained with manageable tolerability profile reinforces the need for wider availability of this novel drug.

## Conclusion

Tazemetostat in INI1-negative ES serves as an example of personalized medicine in the treatment of soft tissue sarcomas. Further, confirmatory trials and long-term follow-up will provide better insight into the efficacy of this drug. Armed with the major advantages of being an oral, targeted agent with manageable toxicity profile this drug will serve to benefit patients with advanced disease.

## Future perspective

The success of tazemetostat in recently published literature has provided an encouraging avenue for the treatment of ES. There is an ongoing search for novel pathways to target and treat refractory tumors and epigenetic modifiers are an emerging modality in the field of personalized medicine. The clinically meaningful results with tazemetostat have now led to further studies that are combining it with conventional therapies such as doxorubicin in first-line setting. Longer follow-up and larger studies in the future will further establish its role as a newer targeted therapy in rare diseases like ES.

**Executive summary**

**Background**

- Epithelioid sarcoma (ES) is a rare, slow-growing sarcoma commonly found in young to middle aged patients, predominantly male.
- Distal type ES is most common with extremity being the commonest primary site.
- About 90% of ES have INI1/SMARCB1 deficiency, which is diagnosed on INI1 immunostaining.
- INI1 deficiency is also documented in malignant peripheral nerve sheath tumor, malignant rhabdoid tumor, synovial sarcoma, myoepithelial carcinoma and poorly differentiated chordomas.
- Loss of INI1 function by *SMARCB1* gene deletion, mutation or epigenetic modification leads to overactivation of the histone lysine methyltransferase complex PRC2. PRC2 overactivity has been demonstrated to cause cell proliferation and silencing the genes responsible for differentiation by action of its catalytic unit, EZH2.
- Advanced ES has few therapeutic options in form of anthracyclines, gemcitabine-based regimens and pazopanib with limited data on trabectedin, dacarbazine and vinorelbine.
- The role of tazemetostat in advanced ES and refractory follicular lymphoma has been recently discovered in Phase I and Phase II trials leading to accelerated US FDA approval of the drug for unresectable locally advanced or metastatic ES.

**Case summary**

- We report a case of left lower limb ES with inguinal nodal metastases, post multiple surgeries, inguinal nodal dissection, adjuvant radiotherapy, chemotherapy and pazopanib.
- The patient attained clinical and radiological benefit with tazemetostat within 2 months of therapy with no major toxicity and continues to tolerate the drug well.

**Discussion**

- About 95% of ES patients undergo upfront surgical resection and 57% develop local recurrence and have distant metastases in the rest.
- About 10–14% patients of ES have inguinal nodal metastases, which were also found in our patient.
- Local recurrences should be evaluated by MRI to look for intransit and nodal metastases.
- The EZH2-202 trial demonstrated an overall response rate of 15% including complete response 1.6% and disease control rate of 26% at 32 weeks with median time to response of 3.2 months.
- Compared with the grade 3–4 toxicities reported in previous studies comprising of anemia, weight loss, pleural effusion, reduced appetite and cancer pain, our patient had no major toxicity and tolerated the therapy well.
- Newer therapeutic modalities such as tazemetostat and doxorubicin frontline combination, and pembrolizumab are subjects of current ongoing research.
